# Integration of a Gold-Specific Whole *E. coli* Cell Sensing and Adsorption Based on BioBrick

**DOI:** 10.3390/ijms19123741

**Published:** 2018-11-24

**Authors:** Li Yan, Peiqing Sun, Yun Xu, Shanbo Zhang, Wei Wei, Jing Zhao

**Affiliations:** 1 State Key Laboratory of Coordination Chemistry, Institute of Chemistry and BioMedical Sciences, School of Chemistry and Chemical Engineering, Nanjing University, Nanjing 210093, China; yanlijsw@163.com (L.Y.); peiqingsun@163.com (P.S.); mg1830093@smail.nju.edu.cn (Y.X.); 2 State Key Laboratory of Pharmaceutical Biotechnology, School of Life Sciences, Nanjing University, Nanjing 210093, China; 3 College of Food Science and Engineering, Northwest A&F University, Yangling 712100, China; zhangshanbo@nwafu.edu.cn

**Keywords:** whole-cell bacterial biosensor, BioBrick, gold detection, gold adsorption, red fluorescence protein

## Abstract

Detection and recovery of heavy metals from environmental sources is a major task in environmental protection and governance. Based on previous research into cell-based visual detection and biological adsorption, we have developed a novel system combining these two functions by the BioBrick technique. The gold-specific sensory *gol* regulon was assembled on the gold-chaperone GolB (Gold-specific binding protein), which is responsible for selectively absorbing gold ions, and this led to an integration system with increased probe tolerance for gold. After being incorporated into *E. coli*, this system featured high-selective detection and recycling of gold ions among multi-metal ions from the environment. It serves as an efficient method for biological detection and recovery of various heavy metals. We have developed modular methods for cell-based detection and adsorption of heavy metals, and these offer a quick and convenient tool for development in this area.

## 1. Introduction

Gold (Au) is historically a highly valued noble metal. It has been used for centuries by humans with its obvious and has superior properties to those of its heavy metal peers [1,2]. As a result of its high stability and rarity, gold has long been the most significant hard currency. With the exploration of biological and pharmaceutical sciences, the function of gold in these fields has also been revealed and it now enjoys important roles in related endeavors, such as self-assembling materials, anticancer drugs and bacterial resistance [3,4,5,6]. However, unavoidable problems in the mining and industry of gold metals today include the loss of gold resources during processing and a more severe impediment, environmental pollution [7,8,9]. To improve the efficiency of the use of rare gold resources without damaging the environment, convenient and green technology used in the detection and recycling of gold has become a subject of increasing interest from scientists.

Traditional methods for detection of gold ions in the environment are based on its physicochemical properties, and include inductively coupled plasma mass spectrometry (ICP-MS) and atomic absorption spectroscopy (AAS), but these methods are restricted by the cost of apparatus and complexities of sample pretreatment [10,11,12]. Novel methods based on chemicals such as chemopolymers and nanoparticles also have drawbacks such as a limited operating environment and being a cause of secondary pollution [13,14,15]. As a result, cell-based probes for metal ions have been developed [16]. Metal analysis in microorganisms is a proven technique and has the advantages of simplicity, excellent biosecurity and environmental neutrality [17]. 

In recent years, a series of bacteria modified by genetic engineering for whole-cell detection have been used in environments containing various heavy metals [18,19,20]. In 2011, Jouanneau et al. reported four engineered bioluminescent bacteria that were used in detection of Hg, As, Cd and Pb ions with a limit of detection in the nanomolar region [21]. In our previous work, we reported the first example of a whole-cell microorganism probe for selective gold-sensing [22]. This was accomplished by combining the gold-sensory protein GolS, gold-binding chaperone GolB and its up-downstream promoter with a fluorescent protein. The probe has high sensitivity and specificity in detection of gold ions. Its effects are visible to the naked eye, which indicates a convenience superior to that of spectrophotometric methods. Meanwhile, we also constructed a bacteria surface display system for recovery of gold ions via a highly selective gold binding protein-GolB. This showed that engineering of metal specific regulons by synthetic biology could provide an alternative method for effectively dealing with heavy metals recovery.

In this article, we report a unified whole-cell system combining specific detection using the GolS regulon with selective adsorption by the surface displayed GolB protein. This integration was achieved by “BioBrick”, a technique in synthetic biology [23,24] which was introduced in the development of bioremediation for precious metal ions. 

## 2. Results and Discussion

### 2.1. Construction of a Fluorescent Gold Biosensor

**Figure 1:**
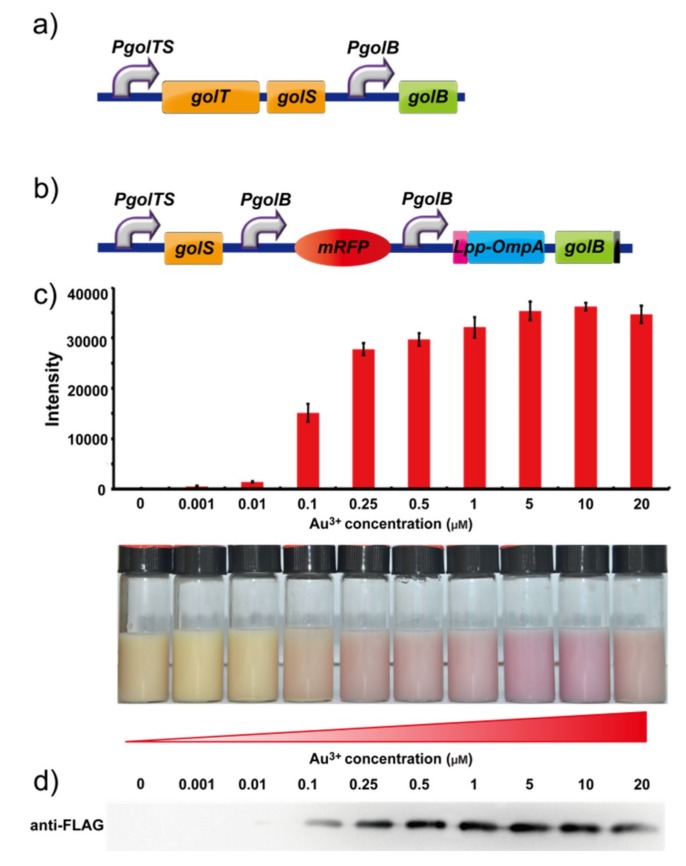
(**a**) Genetic organization of the *gol* locus in the *S. typhimurium LT2* genome. (**b**) Genetic organization of the gold inductive RFP expression plasmid in *E. coli*. (**c**) Fluorescence measurement of *E. coli* cells containing the gold-induced RFP expression plasmid after induction with gradient concentrations of HAuCl_4_ and resuspension in PBS buffer (pH 7.4) and a photograph of the corresponding fluorescence measurement. (**d**) Western blot analysis of the RFP expression under the different concentrations of Au^3+^.

In *Salmonella*, the promoter *pgolTS* began the expression of the protein GolS. When gold ions entered the cell and bonded with protein GolS, the metal protein complexes were bound to the promoter *pgolB* and further modified the expression of GolB (Figure 1a). Inspired by this effect and the results of our previously reported work, we tried to unify the detective and adsorptive system in *E.coli* which is selectively induced by gold ions [22]. The GolB gene is replaced with transformed *rfp* gene after the *golS* promoter in the Biobrick pSB1A2. When gold ions are added to the environment, *rfp* is induced by this system as a signal of the presence of gold. When protein Lpp-OmpA is integrated with GolB as a fusion protein, GolB will be secreted outside the cell, enabling the gold ion to be absorbed on the surface of membrane and recycled in subsequent steps [22,25]. 

In our construction of a carrier protein, we found that simple insertion of an *lpp-ompA-golB* gene into the next position of the initial *rfp* gene failed. Therefore, two *golb* promoters with the same direction were introduced in our designed regulation pathway. The first promoter is responsible for expression of *rfp* in the detection process and the second one is set for protein GolB in recycling. Based on this regulatory mechanism (Figure 1b), the recombinant bacteria were constructed and passed the sequencing test. The novel integrated system for gold ions was completed after transforming the plasmid into *E. coli DH5α*.

### 2.2. Expression of Recombinant Proteins

The RFP was regulated by the *golB* promoter in the BioBricks vector pSB1A2, which allowed the constitutive expression of GolB protein to respond to gold ions in solution and trigger the expression of RFP when transformed in *E. coli* cells. The promoter *pgolB* started the expression of protein GolB. When gold ions were added to the transformed *E. coli*, the metal bonds with protein GolB and further modify the expression of the red fluorescent protein *rfp*. These results demonstrated that even when the induced concentration of gold ions was as low as 0.001 µM (Figure 1c); the red fluorescence of the *E. coli* cells could still be detected by a microplate reader, and are much more sensitive than previously reported bioluminescent bacteria. The red fluorescence of *E. coli* cells was visible at a concentration of 0.1 µM. The results of western blotting shows (Figure 1d) that peak distribution with climax expression of protein appears when concentration of Au^3+^ is 5 μM, which is consistent with the results of the fluorescence intensity analysis (Figure 1c).

### 2.3. Characterization of Time Gradients and Concentration Gradients for Gold Ions

**Figure 2:**
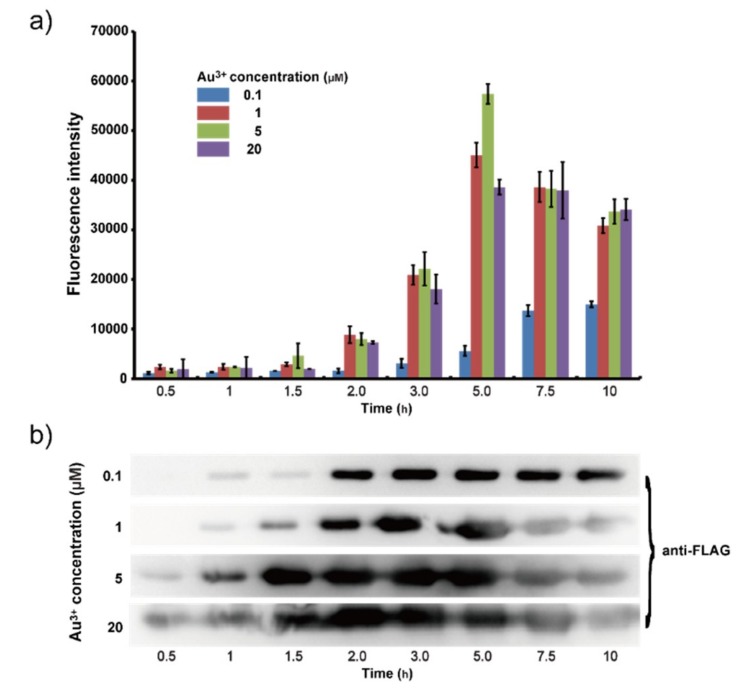
(**a**) Fluorescence measurement of the sensitivity in LB medium containing gradient and time concentrations of Au ions; (**b**) Lpp-OmpA-GolB protein expressed verification.

Time gradients of 0.5, 1, 1.5, 2, 3, 5, 7.5 and 10 h were set at different concentrations of gold ions (0.1, 1, 5 and 20 μM). The results of the fluorescence experiments showed that when the Gold ion concentration was 0.1 μM, the intensity increased with time and reached the maximum value of 16,000 after 10 h (Figure 2a). Western blotting indicated that the maximum expression of protein Lpp-OmpA-GolB was reached after 3 h (Figure 2b). When the concentration of gold ions was 1 μM, fluorescence intensity showed a peak distribution with a maximum of 45,000 after 5 h while the expression of Lpp-OmpA-GolB was maximized after 2 h. This mismatch of fluorescence test and western blotting could be explained as follows. After the saturation of GolB protein on the cell surface, gold ions continuously enter the bacteria inducing the expression of *rfp*. When the concentration was 5 μM, the peak value of two analysis appeared at the same time (5 h), as did the results when the concentration increased to 20 μM.

### 2.4. Characterization of Selectivity for Gold Ions

**Figure 3:**
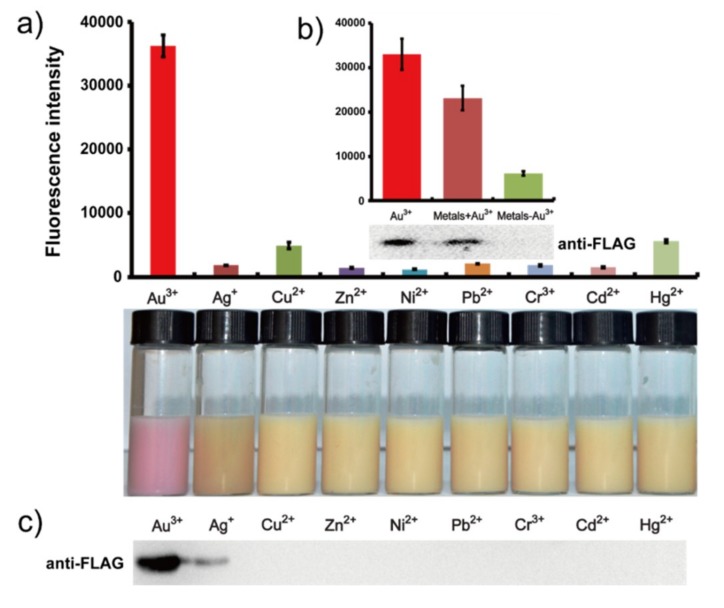
(**a**) Fluorescence measurement of *E. coli* cells containing the gold-induced plasmid expressed by recombined proteins with concentrations of 10 μM of metal ions and a photograph of the corresponding fluorescence. (**b**) Fluorescence measurement of the selectivity to mixed metal ions (Au^3+^, Ag^+^, Cu^2+^, Zn^2+^, Ni^2+^, Cd^2+^, Cr^3+^, Hg^2+^ or Pb^2+^). (**c**) Lpp-OmpA-GolB fusion protein expressed verification by Western blotting using anti-FLAG antibody. The first lane is the Au^3+^ induction case. Figure 3a,c, consistent on the each of the upper and lower lane.

When our whole-cell engineered bacteria were applied in both the separate (Figure 3a) and mixture systems (Figure 3b), the results of fluorescence intensity showed obvious high selectivity for gold ions, a result also visible to the naked eye (Figure 3a). The expression of protein Lpp-OmpA-GolB was tested in western blotting and also has confirmed this conclusion in the mixture systems (Figure 3c). The expression was influenced slightly by other metal species.

### 2.5. Characterization of the Adsorption of Gold Ions

**Figure 4:**
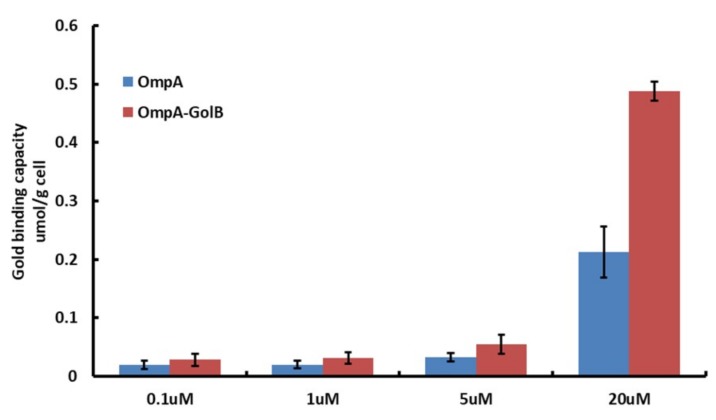
Gold adsorption of surface displayed *E. coli* strains containing OmpA or OmpA-GolB was induced by 20 µM HAuCl_4_ (Au^3+^).

The two GolB-displaying *E. coli* were first incubated for 10 h in LB medium with the concentration of gold ions increasing from 0.1 to 20 μM and then analyzed by ICP-AES after extensive washing. *E. coli* bacteria without induction or expressing only OmpA were used as controls. As shown in Figure 4, compared with the gold binding capacity of bacteria OmpA, the OmpA-GolB is improved. The engineering bacteria all show obvious advantages over the reference bacteria in each trial. In addition, the gold tolerance of *E. coli* cells with or without surface-displayed GolB was measured through a plate sensitive assay. The incubation was operated at 37 °C for 5 h. And, as shown in the result, *E. coli* with surface-displayed GolB protein survived in LB agar plates containing 0–20 μM Au (III) while those without surface-displayed GolB protein showed little tolerance of the environment containing 30 μM Au (III). Our proposed explanation is that gold ions are adsorbed by the surface-displayed GolB protein, consequently improving the gold tolerance of bacteria cells.

## 3. Materials and Methods

### 3.1. Construction of Gold Biosensor

The gold sensing plasmid *gol-rfp*/pSB1A2 and the Lpp-OmpA-GolB fusion protein expression plasmid pBAD, produced with a previously published protocol [22,25] were provided to our laboratory by Dr. Wei Wei of Nanjing University. The gold detecting and adsorptive plasmid was constructed in three steps. First, a DNA fragment including the gene encoding P*golB* was amplified from the *gol-rfp*/pSB1A2. After being confirmed by sequencing, the PCR product was digested by *XbaI* and *PstI* and then inserted the plasmid pSB1A2 and digested by *SpeI* and *PstI*. The gene fragment encoding Lpp-OmpA-GolB was obtained from the *lpp-ompA-golb*/pBAD, and then this DNA fragment was digested by *XbaI* and *PstI*; and inserted into the plasmid which included the modified *gol* regulon. Finally, the plasmid was transformed into the *E.coli* strain DH5α.

### 3.2. Expression of Recombinant GolB Protein from E. coli

To confirm the expression of the red fluorescence protein (RFP), the modified *gol* regulon was expressed in *E. coli* strain *DH5α* with induction by 20 µM of HAuCl_4_ (Au^3+^) with shaking at 37 °C for 10 h. The cells were then harvested and re-suspended in phosphate buffer saline (PBS pH 7.4) and subjected to fluorescence analysis. Fluorescence was recorded using a Multi-Mode Microplate Reader (BioTek, Winooski, VT, USA) with filters at wavelengths of 558 nm for excitation and 583 nm for emission. 

The C-terminal Flag tagged-GolB displayed *E. coli* cells were harvested by centrifugation at 4500 rpm for 5 min then re-suspended in lysis buffer (PBS, pH 7.4). After sonication, the two supernatant fractions and the cell membrane were separated by centrifugation at 4 °C and 11,000 rpm for 15 min. The cell membrane fraction was re-suspended in 100 µL PBS and 100 µL of the supernatant fraction were both mixed with 10 µL of 10× loading buffer then heated at 95 °C for 10 min. After centrifugation at 11,000 rpm for 10 min at 4 °C, the samples were loaded onto 15% SDS-PAGE gels and electrophoresed for 30 min at 80 V and 50 min at 150 V. For Western blotting analysis, the separated proteins were held on polyvinylidene difluoride membranes (BioRad, Solna, Sweden) at 250 mA, at 4 °C for 2 h. After blocking at room temperature for 1 h in Blotto (5% nonfat dry milk in 1× TBST), the membranes were developed using 1:1000 dilutions of monoclonal anti HA-tag (Santa Cruz, CA, USA) or monoclonal anti FLAG-tag (Santa Cruz) as primary antibody for 10 h at 4 °C. This was followed by incubation with horseradish peroxidase (HRP)-conjugated goat anti-mouse secondary antibodies (Santa Cruz) at room temperature for 2 h. Antibodies were detected with ECL reagents (Pierce, Rockford, IL, USA).

### 3.3. Measurements of Gold-Specific Biosensors

We investigated the effect on the engineered bacteria of the concentration and the induction time with gold ions. The *E. coli* strain containing the plasmid was cultured in an LB medium containing ampicillin (50 µg·mL^−1^) until OD600 = 0.6–0.8. The mixture was subjected to a gradient concentration of HAuCl_4_ (Au^3+^) (0.1, 1, 5, 20 μM) with shaking at 37 °C for a gradient induction time (0.5, 1, 1.5, 2, 3, 5, 7.5, 10 h) and then the cells were harvested and normalized to OD600 = 1.0 with PBS buffer (pH 7.4), measured by a Multi-Mode Microplate Reader (BioTek, Winooski, VT, USA) with 558 and 583 nm filters for the excitation and emission wavelengths, respectively.

For the gold concentration sensitivity measurement, the *E. coli* strain containing the plasmid was cultured in LB medium containing ampicillin (50 µg·mL^−1^) until OD600 = 0.6–0.8, then induced by a gradient concentration (0, 0.001, 0.01, 0.1, 0.25, 0.5, 1, 5, 10, 20 μM) of HAuCl_4_ with shaking at 37 °C for 10 h. Meanwhile, the *E. coli* cells were also cultured in an LB medium containing ampicillin (50 µg·mL^−1^) until OD600 = 0.6–0.8. Then a final concentration of 20 μM Au^3+^, Ag^+^, Cu^2+^, Zn^2+^, Ni^2+^, Cd^2+^, Cr^3+^, Hg^2+^ or Pb^2+^ was added to the medium respectively. All of the cells were harvested and normalized to an OD600 = 1.0 with PBS buffer (pH 7.4). Additionally, a mixed metal solution with or without Au^3+^ ions was used to induce the engineered cells as a single metal induction protocol. For fluorescence determinations, a 300 μL aliquot of each sample was applied in triplicate to a 96-well flat bottom black plates (Corning, NY, USA). Fluorescence was recorded using a Multi-Mode Microplate Reader (BioTek, Winooski, VT, USA) with 558 and 583 nm filters for the excitation and emission wavelengths, respectively.

### 3.4. Bioadsorption of Gold by Engineered Cells

The OmpA or OmpA-GolB fusion proteins were expressed in *E. coli* strain *DH10B*. Cells were grown in LB medium containing ampicillin (50 µg·mL^−1^) with shaking for 10 h at 37 °C. After 1:100 dilution in LB medium containing ampicillin (50 µg·mL^−1^), the culture was grown at 37 °C to OD600 = 0.6–0.8. Protein expression was induced by the addition of arabinose to a final concentration of 0.002% and then incubated at 37 °C for 10 h. For gold ion adsorption, 0.1, 1, 5, 20 μM concentrations of gold ions were added to LB medium during the induction of the Lpp-OmpA-GolB fusion proteins. To measure the metal ion adsorption ability of GolB-displayed *E. coli*, the cells were harvested from LB medium by centrifugation at 4000 rpm for 10 min and then washed with double distilled H_2_O at least three times. The gold-adsorbed cells were lyophilized to measure the dry weight and subjected to wet ashing. The samples were then analyzed using an inductively coupled plasma-atomic emission spectrometer (ICP-AES; Optima 5300DV, PerkinElmer Inc., Wellesley, MA, USA).

## 4. Conclusions

Inspired by previous studies of whole-cell biodetection and bioadsorption, this research succeeded in integrating a gold ion selective detection system based on the GolS regulator with the gold ion selective adsorption system based on the surface display of GolB protein using the synthetic “BioBrick” biology technology. The high selectivity and sensitivity towards gold of these engineered *E. coli* cells provides a new way to design novel metal biosensors, and by improving the original resistance to the toxicity of the gold ion in the ion probe of the whole cell, the integration finally achieved a integration of expression system in detection and adsorption of gold ions regulated by the gradients of gold ions in environment. Thus selective detection and adsorption of gold ion from the mixture of several ions was achieved. It should be possible for *B. subtilis* to selectively detect and absorb trace levels of gold ions by homologous recombination of this system, which provides an important direction for detection of other toxic heavy metal ions. This study will make this kind of construction of whole cell detection and adsorption more patterned, and could realize a more convenient method of detection and adsorption of heavy metals.
